# Tai Chi Chuan in postsurgical non-small cell lung cancer patients: study protocol for a randomized controlled trial

**DOI:** 10.1186/s13063-017-2320-x

**Published:** 2018-01-04

**Authors:** Hong Pan, Yingxia Pei, Bingxue Li, Yi Wang, Jie Liu, Hongsheng Lin

**Affiliations:** 1 Department of Oncology, Guang’anmen Hospital, China Academy of Chinese Medical Sciences, Beijing, 100053 China; 2 Department of Oncology, the first affiliated hospital of Zhejiang Chinese Medical Hospital, Zhejiang, 310006 China; 3 Clinical Medical College, Beijing University of Chinese Medicine, Beijing, 100029 China

**Keywords:** Tai Chi Chuan, Exercise capacity, Non-small cell lung cancer, Postoperation

## Abstract

**Background:**

Impairment of exercise capacity remains a common adverse effect of non-small cell lung cancer (NSCLC) survivors after surgery. Previous research has suggested that Tai Chi Chuan (TCC) offers an exercise capacity benefit in several types of cancers. This is a randomized trial to investigate the efficacy and safety of TCC in postoperative NSCLC patients over an observation period of 3 months and a 9-month follow-up.

**Methods/design:**

Using a prospective, one center and randomized design, 120 subjects with histologically confirmed stage I–IIIA NSCLC following complete surgical resection will potentially be eligible for this trial. Following baseline assessments, eligible participants will be randomly assigned to one of two conditions: (1) TCC training, or (2) placebo control. The training sessions for both groups will last 60 min and take place three times a week for 3 months. The sessions will be supervised with target intensity of 60–80% of work capacity, dyspnea, and heart rate management. The primary study endpoint is peak oxygen consumption (VO_2peak_), and the secondary endpoints include: 6-min walk distance (6MWD), health-related quality of life (HRQoL), lung function, immunity function, and the state of depression and anxiety. All endpoints will be assessed at the baseline and postintervention (3 months). A follow-up period of 9 months will be included. The main time points for the evaluation of clinical efficacy and safety will be months 3, 6, 9, and 12 after enrollment.

**Discussion:**

This study will assess the effect of group TCC in postsurgery NSCLC survivors on VO_2peak_, lung function, and other aspects. The results of this study will eventually provide clinical proof of the application of TCC as one kind of exercise training for patients across the entire NSCLC continuum, as well as information on the safety and feasibility of exercise.

**Trial Registration:**

Chinese Clinical Trial Registry: ChiCTR-IOR-15006548. Registered on 12 June 2015.

**Electronic supplementary material:**

The online version of this article (doi:10.1186/s13063-017-2320-x) contains supplementary material, which is available to authorized users.

## Background

Lung cancer is a prevalent issue worldwide. Data from 2011 indicated that lung cancer, comprising 18.6% of the total new cases and 13.6% of the total deaths, is the most frequently diagnosed cancer in males and the leading cause of cancer death for each sex in both economically developed and developing countries [1]. With a 5-year survival of 14%, it was estimated that about 159,260 Americans would die from lung cancer in 2014 [2]. Lung cancer is histologically categorized into small cell lung cancer (SCLC) and non-small cell lung cancer (NSCLC) (approximately 85% of those diagnosed with lung cancer), and the survival of patients with the latter is much higher than those diagnosed with SCLC [3].

Surgical removal remains the best curative option for patients with early-stage NSCLC and for appropriately selected patients with locally advanced disease. Approximately 26,000 individuals per year in the United States will survive more than 5 years after initial diagnosis of operable disease [4]. With the government’s focus on the early detection of lung cancer screening, an increasing number of patients with early-stage disease were detected, thus elevating the number of curable and surgical resection cases [5]. Despite the improvement in surgical techniques and the survival rate, surgery is still associated with significantly reduced exercise capacity, which in turn is associated with increased morbidity [6, 7] along with functional limitations, decreased health-related quality of life (HRQoL), and psychiatric issues [8].

Clinicians are paying more and more attention to improving overall physical and emotional function as well as longevity. A growing interest has arisen in the use of non-pharmacological interventions such as exercise, which has been identified as a safe and effective way to improve aerobic capacity, strength, body composition, and HRQoL, as well as to reduce fatigue, emotional distress, and lymphedema symptoms in diverse cancers [9–14]. To our knowledge, preliminary data suggest that exercise intervention compared with usual care is associated with improved exercise capacity for postsurgery patients [15]. Nevertheless, the results have been conflicting and the optimal program is still to be determined, such as the type of exercises [16].

Tai Chi Chuan (TCC) is a traditional Chinese exercise oriented in thousands of years of ancient history. It combines meditation with slow and gentle movements, as well as deep breathing to move vital qi throughout the body. It is considered as a complex, multicomponent intervention that involves physical, psychosocial, emotional, spiritual, and behavioral elements [17]. The results of a systematic review including 1868 participants suggested that TCC has a positive effect on the majority of outcomes of exercise capacity including peak oxygen consumption (VO_2peak_) [18]. Furthermore, a recent meta-analysis that pooled the effect of 12 controlled trials examining the efficacy of TCC to improve the cancer-specific HRQoL reported that the weighted mean difference was 7.99 (95% confidence interval (CI) 4.07–11.91; *Z* score = 4.00, *p* < 0.0001) [19]. In addition, compared with conventional exercises, research among 61,477 adults revealed TCC as the most common forms of exercise among Chinese adults and is related to reduced cancer-caused mortality [20]. Meanwhile, as a mind-body technique, TCC has been found to better lower mental disorder and fall rate in cancer survivors relative to conventional therapeutic exercise [21]. Given the clinical benefits of TCC, none of these studies evaluated the influence of TCC among postoperative NSCLC patients.

In light of this evidence, we designed a single-blind randomized controlled trial to evaluate the efficacy and feasibility of TCC on postoperative NSCLC patients. We hypothesize that TCC may serve as a safe exercise intervention that offers potential benefit among postsurgical NSCLC survivors.

## Methods

### General objective

To investigate the efficacy and feasibility of TCC on postoperative NSCLC patients with the aim of confirming the effectiveness and safety of TCC.

### Study design and procedures

**Fig. 1:**
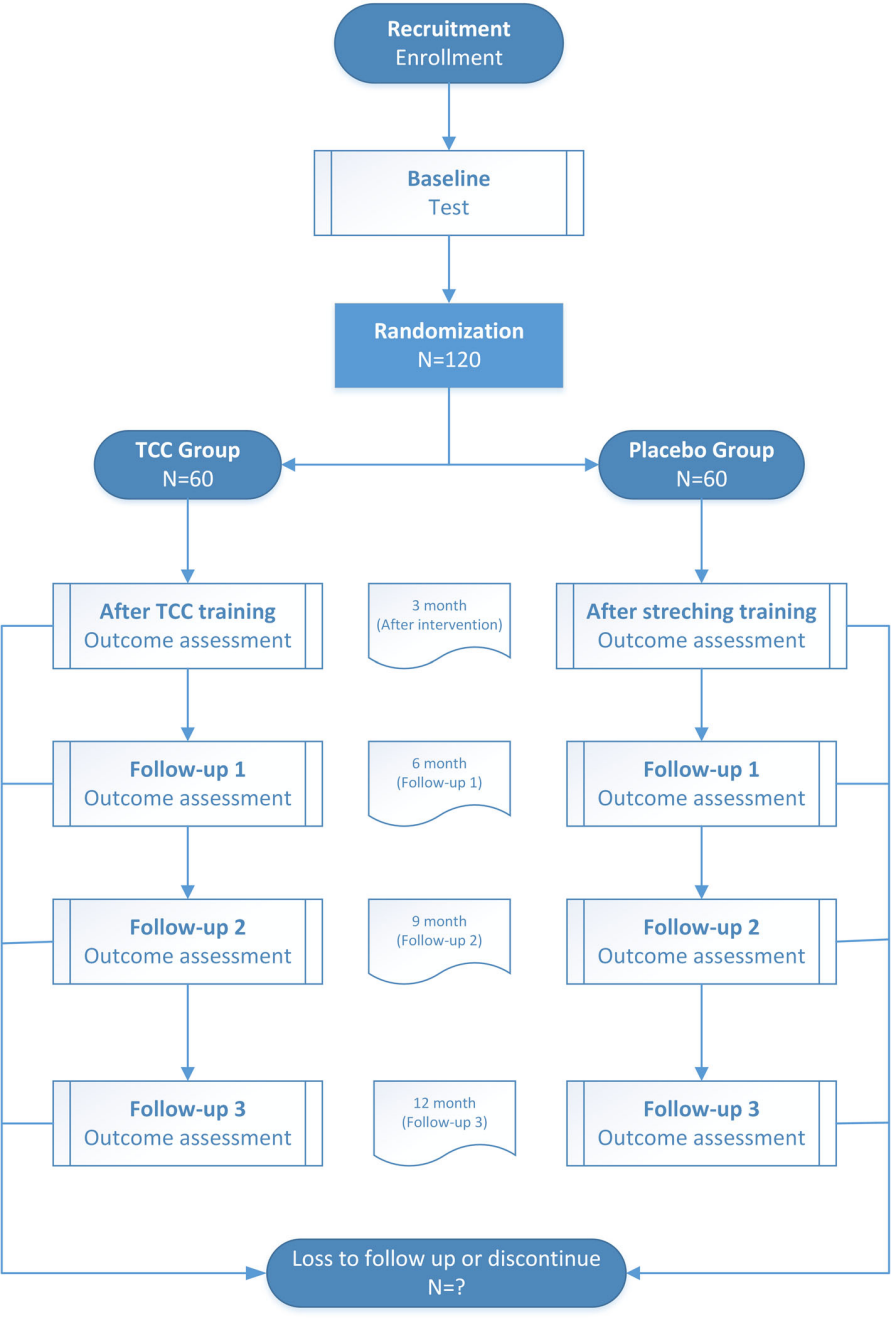
Flow diagram. *TCC* Tai Chi Chuan

The study will be a prospective, single blind, parallel group, randomized controlled trial. We will recruit and randomize 120 histologically confirmed stage I–IIIA postsurgical NSCLC patients 1:1 into two groups: 1) TCC training; or 2) placebo control. The study period will be 12 months including a 3-month supervised exercise training course and a 9-month follow-up course with the first and secondary outcomes measured at baseline, and at 3, 9, and 12 months, along with other assessments. The procedure is shown in Fig. 1. All patient visits, exercise, and assessment will take place in Guang’an men Hospital, China Academy of Chinese Medical Sciences, Beijing, China. This report will be compiled in line with the SPIRIT (Standard Protocol Items: Recommendations for Interventional Trials) 2013 Statement “Defining Standard Protocol Items for Clinical Trials” [22]. Additional file 1 shows this in more detail.

### Participants

This study will be conducted in the Department of Oncology, Guang’an men Hospital, China Academy of Chinese Medical Sciences. Each member of staff of the study team will be able to enroll participants. Inclusion criteria will be as follows: 1) histologically diagnosed with Stage I–IIIA NSCLC according to the 7th edition cancer staging manual of the American Joint Committee on Cancer [23] and had received surgical therapy; 2) had survived no more than 6 months since surgery; 3) Karnofsky performance status of at least 70% at study entry; 4) estimated life expectancy ≥ 6 months; and 5) aged between 18 and 65 years. All study subjects will meet all five inclusion criteria.

Exclusion criteria will be as follows: 1) subjects with serious systemic diseases, such as cerebrovascular, liver, kidney, hematopoietic system, and psychiatric diseases; 2) unable to participate in an exercise training because of either bone or joint diseases; 3) patients who are in other clinical trials; and 4) unable to understand Chinese. Every enrolled patient will sign an informed consent (seen in Additional file 2) before taking part in the later project procedures.

Measures to guarantee adequate participant enrolment will include: 1) a free rehabilitation and exercise training program; 2) the offer of priority access to specialist consultation; and 3) additional general guidance and disease consultation.

### Randomization and blinding

After completing baseline testing, each participant will be randomly allocated in a 1:1 ratio to either the TCC group or the placebo group. Each participant will learn of their group assignment by receiving a sequentially numbered and sealed opaque envelope from the project director and opening the envelope that contains the group assignment randomly assigned to their sequence number as randomly generated by the computer. Measurement technicians who are responsible for data collection and coding data are blinded to participant group assignment. In addition, all study personnel conducting the study assessments at baseline and postintervention will be blinded to treatment assignment for the duration of the study.

### Study interventions

Participants in each group will attend a supervised 1-h exercise session three times per week, starting 4 weeks after surgery at the earliest. A senior exercise physiologist will conduct the exercise session. Each session includes 10 min low-intensity warming up (50–60% of maximum heart rate) and 5 min cooling down at the beginning and end of the course, respectively. All interventions will be tailored to each participant following the principles of aerobic or resistance training prescription guidelines for adults as recommended by the American College of Sports Medicine (ACSM) [24]. The Borg rating of perceived exertion (RPE) and blood pressure will be assessed prior to and after the exercise, with heart rate and O_2_ saturation monitored continuously throughout.

#### TCC training

The TCC training group will learn to perform 24 standard TCC movements and breathing techniques taught by a professional TCC master with more than 20 years of teaching experience. The instructor explains the theory behind TCC and provides printed materials about TCC when the patient is placed in this group. In each session, participants practice TCC under the master’s instruction. Each session includes a 10-min warm-up about self-massage, breathing techniques, and relaxation in TCC, 45 min exercise training and 5 min cooling down. Throughout the intervention period, participants are required to practice TCC for 20 min every 4 days at home. After 3 months, participants are encouraged to practice TCC using an instructional DVD and keep record of activities until the follow-up visit at month 12.

#### Placebo control

Participants in the placebo control group will attend a supervised stretching program of the same frequency, duration, and length as the TCC group. The stretching program will be prescribed according to ASCM guidelines for people aiming to increasing whole-body flexibility. Stretches will be performed from a seated position in order to minimize weight-bearing forces. The stretching will also be limited in duration to 45 min, alternating between lower and upper body muscle groups in combination with warm-up and cooling down under the supervision of a professional physiologist. Throughout the intervention period, participants will be asked to stretch for 20 min every 4 days at home. After 3 months, participants will be encouraged to do stretching until the follow-up visit at month 12 and keep a record.

### Adherence considerations

Participants will be encouraged to accomplish the routine exercise over the 3 months. Several strategies will be employed to maximize adherence including individualized attention at the intervention sessions and telephone calls following missed sessions. In addition, participants who miss a session will be asked to attend a make-up class. Furthermore, we set the same classes in the morning and afternoon for patients to choose from, which promotes attendance by improving accessibility. Thirdly, all the participants will be asked to complete daily logs about the amount of time they practiced TCC or stretches. Finally, the study staff will also meet on a weekly basis to review each participant’s adherence.

### Study endpoints and assessments

#### Primary endpoint

##### VO_2peak_

VO_2peak_ will be evaluated using a physician-supervised incremental cycle ergometer test with 12-lead electrocardiogram (ECG) monitoring (Mac® 5000, GE Healthcare) under the supervision of a physiologist blinded to the patient’s randomization group. According to prior studies by Jones et al. [25, 26], all the subjects will pedal at 20 W for 1 min, and increase by 5 W every minute until exhaustion or a symptom-limited VO_2peak_ is achieved. Exercise will be terminated if any ECG abnormalities are observed.

#### Secondary endpoints

The secondary endpoints of this trial will be the 6-min walk distance (6MWD), lung function, patient-reported outcomes, and immunity function.

##### 6MWD

6MWD is a useful measure of functional capacity, which has been widely used for postoperative evaluation and for measuring the response to therapeutic interventions for pulmonary disease. The test will be performed according to current guidelines [27] in a 30-m corridor. Patients will be instructed to walk at their fastest pace and cover the longest possible distance in 6 min; pauses are allowed if needed. Waiting at least 1 h before a second test, the highest 6MWD is reported. Measurement of peripheral oxygen saturation and heart rate will be performed before and immediately after the test. Participants were asked to rate their post-test dyspnea and fatigue level using the Borg scale (rate 6–20) [28], with a higher rating corresponding to higher dyspnea or fatigue level.

##### Lung function

Lung function was found to be significantly associated with postoperative mortality in NSCLC, especially the percentage of forced expiratory volume in 1 s (FEV1) [29, 30]. This parameter will be evaluated using a calibrated spirometer (Spirovit SP-2; Schiller, Swizerland). FEV1 and forced vital capacity (FVC) will be measured, and FEV1/FVC will be calculated and compared to reference values [31]. The patients will be measured three times and the best result will be recorded.

##### Patient-reported outcomes

Patient-reported outcomes will include HRQoL, fatigue, dyspnea, depression, and anxiety. The 42-item Functional Assessment of Cancer Therapy–Lung (FACT-L) scale, which contains four subscales for physical (seven items), functional (seven items), emotional (six items), and social/family (seven items) well-being, plus a lung cancer-specific subscale (15 items), will be used to assess patient symptoms and HRQoL [32]. The 13-item FACT—fatigue scale will be used as the assessment of fatigue [33]. The Cancer Dyspnea Scale (CDS), which has been demonstrated to be a valid and feasible scale for assessing cancer-related dyspnea among NSCLC patients [34, 35], will be used to evaluate dyspnea [36]. Finally, depression and anxiety will be assessed using the Self-rating Depression Scale (SDS) and the Self-rating Anxiety Scale (SAS). All the above instruments have been found to be easy to administer, widely used, and reliable in prior research in lung cancer patients.

##### Immunity function

Immunity function will be tested by using peripheral blood samples collected at 7:00 am at baseline, at completion of the 3-month study, and at 9 months follow-up. Flow cytometry (Beckman Coulter EPICS XL; Beckman Coulter, Inc., Brea, CA, USA) will be used for the analysis within 24 h of collection of the blood samples. The percentage of T lymphocytes, CD4^+^ and CD8^+^ T lymphocytes, and the ratio of T helper/T suppresser cells will be used for the assessment.

### Tracking and monitoring of adverse events

Several methods will be used to assure the tracking and monitoring of adverse events: 1) all patients will be under the supervision of an oncologist and every single adverse event will be recorded on a case report form (CRF); 2) at the beginning of the training intervention, the physiologist will discuss the potential side-effects of the exercise, and all episodes occuring during the class will be recorded on the CRF; 3) during the exercise course and assessment, the blood pressure, heart rate, and SpO_2_ will be monitored in case of any negative side-effect; 4) any severe adverse event will be reported to the monitor of the trial and Guang’an Men Hospital within 24 h.

**Fig. 2:**
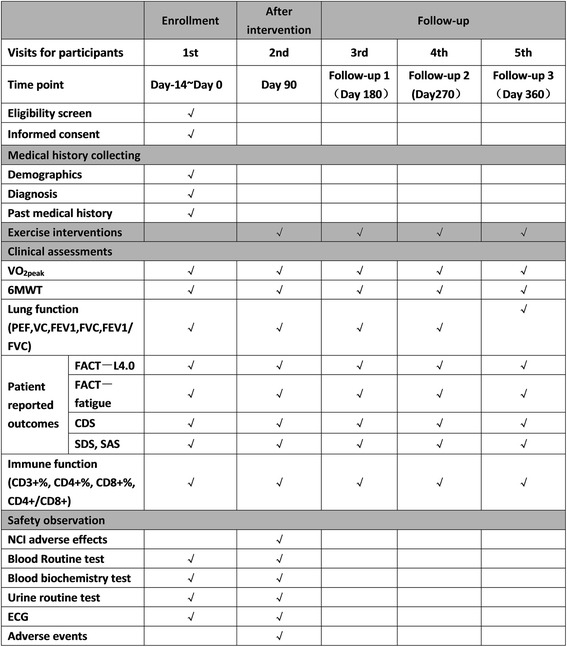
Time schedule of enrollment, interventions, and assessments, as well as visits for participants. *6MWT* 6-min walk test, *CD* cluster of differentiation, *CDS* Cancer Dyspnea Scale, *ECG* electrocardiograph, *FACT* Functional Assessment of Cancer Therapy, *FEV1* forced expiratory volume in 1 s, *FVC* forced vital capacity, *NCI* National Cancer Institute, *PEF* parameter peak flow, *SAS* Self-rating Anxiety Scale, *SDS* Self-rating Depression Scale, *VO*_*2peak*_ peak oxygen consumption

The time schedule of the study, including enrollment, interventions, assessments, and visits for participants, is shown in Fig. 2.

### Data management and monitoring

A specialized clinical research associate (CRA) will be responsible for checking the ranges for data values. Double data entry will be conducted to promote data quality. Operations and data will be audited at the end of the trial by the state administration of traditional Chinese medicine, which is the department that oversees the project. Clinical Research Organizations (CRO), which are independent from investigators and the sponsor, will monitor the data during every participant visit.

### Statistical analysis

This randomized trial will recruit 120 subjects with postsurgical NSCLC. Unpaired *t* tests will be used to analysis the primary and secondary endpoints by comparing the TCC to the placebo group regarding mean improvement. We assume that improvement in VO_2peak_ in the TCC group will be 22.5%, while it will be 5% in the placebo group, as reported previously [15]; thus, a minimum of 55 patients per group will be required. Accounting for a 10% dropout rate, the inclusion of 60 patients per group results in a total of 120 patients.

The principal analysis of the study endpoints will employ the intention-to-treat (ITT) approach. The ITT analysis will include all randomized participants in the random allocation. The intervention group assignment will not be altered based on the participants’ adherence to the randomly allocated study arm. Patients who are lost to follow-up will be included in all primary and secondary analyses by assuming zero change across time. For the primary analysis, a multiple regression model will be used to regress change in VO_2peak_ on study group, the baseline value of the endpoint, and other pertinent baseline variables that may influence change in the study endpoints (e.g., co-morbid conditions/medications, self-reported exercise history, age).

All statistical analysis will be conducted using SPSS 17.0 statistics software. The data of all the endpoints are quantitative data. The mean ± standard deviation will be used to describe symmetric data validated by distribution pattern and homogeneity of variance, otherwise the interquartile range (IQR) will be used. If the data are symmetric after being validated by distribution pattern and homogeneity of variance, a *t* test will be used to compare the differences between the quantitative indices between the groups, otherwise the Wilcoxon rank-sum test will be used. For each endpoint, the overall alpha level will be controlled at a one-sided 0.05 by using Holm’s procedure [37]. The collected data will be examined, and the researchers will be supervised to control bias.

## Discussion

Surgical removal is the best curative option for patients with early-stage NSCLC and for appropriately selected patients with locally advanced disease. Increasing numbers of patients are eligible for receiving surgical intervention [5]. Despite the possibility of a cure, NSCLC survivors with lung resection surgery are often deconditioned and may have varying degrees of impairment of exercise capacity [38] and HRQoL [39] due to the unique pathophysiology of NSCLC, conventional therapeutic strategy, and physical inactivity. Additionally, studies conclude that operable NSCLC patients are at a higher risk of developing emotional distress than other diagnosis groups [40]. Surgically treated NSCLC patients are reported to suffer from persistent physical functional impairment lasting up to 12 months after surgery or longer [41]. Recently, epidemiologic studies and findings suggest that regular exercise can decrease the cancer-related mortality and all-cause mortality among cancer survivors [42], and has been identified as a successful intervention to improve physical and psychological health in some cancer populations (mainly breast cancer) [43]. In light of the increase in the diagnosis of people with curable lung cancer, with the likelihood of an anticipated changing landscape of cancer care, exercise intervention for this population needs to be explored urgently.

TCC, a traditional Chinese exercise, has gained a lot of attention in clinical and research applications as a rehabilitation method for cancer survivors. Recent systematic reviews have demonstrated that TCC may have enormous potential as an effective and feasible rehabilitation intervention to improve tolerance of exercise capacity, symptoms, and immunity function for cancer survivors [18, 19]. In addition, TCC is one of the most common forms of exercise among Chinese adults [20] indicating that there is a large potentially eligible population that would be able to be recruited in to the trial.

The main outcome of the present trial is exercise capacity. The gold standard for measurement of exercise capacity is a direct measure of VO_2Peak_; other testing methods include the 6MWD, shuttle walk test, and stair-climbing test. Among these, VO_2peak_ and 6MWD are the two most frequently used (52% and 16%) [44]. Furthermore, VO_2Peak_ is an independent predictor of all-cause pulmonary morbidity [45, 46]. Brunelli and colleagues found that a VO_2Peak_ of no less than 20 mL/kg/min is a safe cutoff value, and no mortality was observed above this threshold for major lung resection [47]. Consistent with this, the findings of Jones et al. suggest that higher VO_2peak_ was associated with a statistically significant 21% to 24% reduction in the risk of mortality, and a VO_2peak_ of < 14 mL/kg/min may identify patients with poor prognosis [48]. Together, this indicates that VO_2peak_ is an emerging evaluating parameter of exercise training in patients with NSCLC. Specifically, research has shown TCC to be beneficial to the VO_2peak_ of middle-aged subjects [49, 50]. Despite the wealth of evidence, research has seldom explored the efficacy of TCC against exercise capacity or VO_2peak_. Thus, the primary outcome of this trial will be VO2peak. 6MWD and lung function will be set as simple and alternative elements to comprehensively assess the exercise capacity of this specific population.

Patients with operable NSCLC also experience fatigue and dyspnea, and are at risk for developing psychosocial problems, reduced quality of life as well as immune function. And, existing evidence have suggested that TCC has a promising function on enhancing proliferative and cytolytic activities of peripheral blood mononuclear cells (PBMCs), as well as increasing the ratio of T helper/T suppressor cells and mediating Th1/Th2 T-helper cell balance in postsurgical NSCLC patients, thus improving the immunity function of the patients [51, 52]. Therefore, our study also takes above symptom and immunity parameters into consideration to comprehensively evaluate the clinical benefit of TCC as an exercise intervention.

To enable TCC intervention to be integrated into clinical practice, it is important to determine the optimal design of TCC intervention that will be feasible, acceptable, and positively affect outcomes in a healthcare setting as rehabilitation in postsurgical patients with NSCLC in a randomized clinical trial. To our knowledge there are currently no clinical trial focusing on TCC intervention for surgically treated NSCLC patients. Therefore, the results of this study will offer an overview of the possible effects of TCC and address many critical unknown questions in order to set the stage for more clinical trials using traditional Chinese exercises such as Qigong and Baduanjin. In the long term, we are looking forward to making a contribution to the establishment of exercise interventions as a non-pharmacologic therapeutic guideline for NSCLC patients.

While the trial will potentially significantly contribute to these fields of research, potential limitations of the study should also be recognized. One difficulty encountered in the design of the trial was selecting an appropriate control condition. There were several options for control participants including: 1) strength training; 2) conventional aerobic exercise; and 3) stretching placebo. Although the first two of these have shown clinical benefit for NSCLC, neither of them was selected. Strength training was not selected because it is too vigorous and unacceptable for NSCLC based on our prior oral pilot questionnaire. Furthermore, the target of the trial is to explore the clinical efficacy and feasibility of TCC, rather than the comparison between TCC and aerobic exercise; thus, stretching as a more tolerable and safe control was selected.

## Trial status

The study will end in 2017. To date, all 120 patients have been recruited.

## Additional file

Additional file 1: SPIRIT 2013 Checklist: recommended items to address in a clinical trial protocol and related documents. (DOC 128 kb)
Additional file 2: Informed Consent form - Informed notice page (Chinese version). (DOC 44 kb)

## Abbreviations

6MWD: Six-minute walk distance
ACSM: American College of Sports Medicine
CDS: Cancer Dyspnea Scale
CI: Confidence interval
CRA: Clinical Research Associate
CRF: Case report form
CRO: Clinical Research Organization
ECG: Electrocardiogram
FACT-L: Functional Assessment of Cancer Therapy—Lung
FEV1: Forced expiratory volume in 1 second
FVC: Forced vital capacity
HRQoL: Health-related quality of life
IQR: Interquartile range
ITT: Intention-to-treat
NSCLC: Non-small cell lung cancer
PBMC: Peripheral blood mononuclear cell
RPE: Rating of perceived exertion
SAS: Self-rating Anxiety Scale
SCLC: Small cell lung cancer
SDS: Self-rating Depression Scale
TCC: Tai Chi Chuan
VO_2peak_: Peak oxygen consumption
