# Supplementary material for: Tai Chi Chuan in postsurgical non-small cell lung cancer patients: study protocol for a randomized controlled trial
Source: Trials. 2018 Jan 4;19:2. doi: 10.1186/s13063-017-2320-x (PMC5753515; doi:10.1186/s13063-017-2320-x)
Supplement: Additional file 2: — Informed Consent form - Informed notice page (Chinese version). (DOC 44 kb) [file 13063_2017_2320_MOESM2_ESM.doc]

知情同意书·知情告知页

尊敬的女士/先生：

我们将邀请您参加一项“中医药行业科研专项-肺癌中医临床指引的示范与推广”康复阶段的研究。

在您决定是否参加这项研究之前，请尽可能仔细阅读以下内容，它可以帮助您了解该项研究以及为何要进行这项研究，研究的程序和期限，参加研究后可能给您带来的益处、风险和不适。如果您愿意，您可以请您的主管医生给予解释，也可以和您的亲属、朋友一起讨论，帮助您做出决定。

研究介绍

　　一、研究背景和研究目的

1．研究背景：项目承担单位前期经过国家六五、七五、八五、九五、十五科技攻关计划课题，取得了中医药参与治疗在非小细胞肺癌治疗应用的循证医学依据；然后在中医局的领导下，整合优化全国的优势力量，制订了中医治疗肺癌的综合治疗方案，即《肺癌中医临床指引》（以下简称《指引》），通过国家十一五支撑计划课题“非小细胞肺癌中医综合治疗方案的研究”的实施，对这个能体现中医综合治疗优势的方案进行队列研究，研究结果提示，中医参与或中医为主的综合治疗方案在肺癌的治疗中具有明显的优势。

本项目在前期研究的基础上，进行创新性的《指引》的转化研究，提供较大样本的《指引》推广效果评估研究数据，明确《指引》的可操作性和优越性，最终实现完善后的《指引》在示范区的全面推广，为今后《指引》在全国范围的推广提供前提和依据。

2．研究目的：评价肺癌康复治疗阶段中医综合方案中规范化传统中医运动对NSCLC术后患者的康复作用。

本研究将在全国5所医院进行，预计有80名受试者自愿参加。

本项研究已经得到中华人民共和国科技部批准。中国中医科学院广安门医院伦理委员会已经审议此项研究是遵从赫尔辛基宣言原则，符合医疗道德的。

　　二、哪些人不宜参加研究

1.有严重、未控制的器质性病变或感染，如失代偿的心、肺、肾功能衰竭等患者；

2.妊娠期或哺乳期妇女，精神病患者；

3.有肢体功能障碍，不能参加运动锻炼者；

4.正在其它临床试验中；

5.对研究药物过敏者。

三、如果参加研究将需要做什么

1．在您入选研究前，您将接受以下检查以确定您是否可以参加研究：

医生将询问、记录您的病史，对您进行体格检查。您需要做血常规、大便常规、尿常规，肝肾功能，肿瘤标志物，免疫功能等理化检查，以及心电图，CT等影像学检查。

2．若您以上检查合格，将按以下步骤进行研究：

主管医生根据患者的诊断、临床辨证及患者的意愿，将分为两组进行观察：观察组和中医组。

观察人群：非小细胞肺癌术后IIIA期完成了3-4个月的术后辅助化疗的患者；不需要做术后辅助治疗的术后1月以上的I-II期患者；

对照组：不予特殊处理，随访观察

传统运动组 ：规范化中医传统运动。

治疗期：进行1个周期（3月）的观察，后随访至1年。3个月复查一次。

在治疗1个疗程后：您应如实向医生反映病情变化，并将您的病史及理化及影像学检查结果复印件提交给医生（住院患者不用提交复印件）。

3．需要您配合的其他事项：

您需要按医生和您约定的随访时间来医院就诊。您的随访非常重要，因为医生将判断您接受的研究措施是否真正起作用。

您需要按医生指导用药，并请您在每次服用免费药后及时、客观地在《服药记录卡》中记录。您在每次随访时都必须归还未用完的药物及其包装，并将正在服用的其它药物带来。

在研究期间您不能使用治疗非小细胞肺癌的偏方验方等非科学治疗方法。如您需要进行其它治疗，请事先与您的医生取得联系。

四、参加研究可能的受益

您和社会将可能从本项研究中受益。此种受益包括您的病情有可能获得改善，以及本项研究帮助形成一种规范的治疗方案。您将在研究期间获得良好的医疗服务。

五、参加研究可能的不良反应、风险和不适、不方便

根据以往的研究结果，传统中医运动耐受性较好，可能出现的手术伤口不适，疲乏，一过性疼痛加重等，一般不影响继续治疗。尽管已经了解很多，治疗中仍还有可能发生不可预知的不良反应。医生会严密观察您的病情变化，如实地告诉您研究过程中的出现的各种现象，积极处理相关不良反应，以利于您的安全。此研究中所用的方案和药物均为规范、疗效可靠、专家共识性治疗，在临床中已经较为广泛的应用，药物不良反应可参照相应的《药品说明书》。

如果在研究中您出现任何不适，或病情发生新的变化，或任何意外情况，不管是否与药物有关，均应及时通知您的医生，他/她将对此作出判断和医疗处理。

医生和课题研究单位将尽全力预防和治疗由于本研究可能带来的伤害。如果在临床研究中出现严重不良事件，医学专家委员会将会鉴定其是否与研究方案有关，如果经确认有关，课题研究单位可提供相应补偿。

您在研究期间需要按时到医院随访，做一些理化检查，这些都是病情需要做的检查以了解您治疗的安全及效果，请您配合。

此外，研究可能出现无效的情况，以及因治疗无效或者因合并其他疾病等原因而导致病情继续发展。在研究期间，如果医生发现本项研究所采取的研究措施无效，将会中止研究，改用其他可能有效的治疗措施。

六、有关费用

本研究将提供免费传统中医运动小班教学及教学光盘，研究及随访期间的与研究有关的诊疗费用由您自理，具体费用与您平时住院/门诊治疗相似，本项目研究不额外增加您的费用。如果您同时合并其他疾病所需的治疗和检查，费用自理。

七、个人信息是保密的吗？

您的医疗记录（研究病历、检查报告等）将完整地保存在医院，医生会将检查结果记录在您的门诊病历上。研究者、研究承担单位的代表、伦理委员会和上级管理部门将被允许查阅您的医疗记录。任何有关本项研究结果的公开报告将不会披露您的个人身份。我们将在法律允许的范围内，尽一切努力保护您个人医疗资料的隐私。

除本研究以外，有可能在今后的其他研究中会再次利用您的医疗记录和病理检查标本。您现在也可以声明拒绝除本研究外的其他研究利用您的医疗记录和病理标本。

八、可以自愿选择参加研究和中途退出研究

是否参加研究完全取决于您的自愿。您可以拒绝参加此项研究，或在研究过程中的任何时间退出本研究，这都不会影响您和医生间的关系，都不会影响对您的医疗或有其他方面利益的损失。

您的医生或研究者出于对您的最大利益考虑，可能会随时中止您参加本项研究。

如果您因为任何原因从研究中退出，您可能被询问有关您使用研究方案的情况。如果医生认为需要，您也可能被要求进行实验室检查和体格检查。这对保护您的健康十分有利。

九、现在该做什么？

是否参加本项研究由您自己决定。您可以和您的家人或者朋友讨论后再做出决定。在您做出参加研究的决定前，请尽可能向您的医生询问有关问题，直至您对本项研究完全理解。

感谢您阅读以上材料。如果您决定参加本项研究，请告诉您的医生或研究助理，他/她会为您安排一切有关研究的事务。

请您保留这份资料。

知情同意书·同意签字页

项目名称：中医药行业科研专项-肺癌中医临床指引的示范与推广

项目编号：201307006

方案编号：HYZX-5-11-KD

承担单位：中国中医科学院广安门医院

伦理审查单位：中国中医科学院广安门医院伦理委员会

伦理批号：

同意声明：

我已经阅读了上述有关本研究的介绍，而且有机会就此项研究与医生讨论并提出问题。我提出的所有问题都得到了满意的答复。

我知道参加本研究可能产生的风险和受益。我知晓参加研究是自愿的，我确认已有充足时间对此进行考虑，而且明白：

- 我可以随时向医生咨询更多的信息。
- 我可以随时退出本研究，而不会受到歧视或报复，医疗待遇与权益不会受到影响。

我同样清楚，如果我中途退出研究，特别是由于药物的原因使我退出研究时，我若将病情变化告诉医生，完成相应的体格检查和理化检查，这将对我本人和整个研究十分有利。

如果因病情变化我需要采取任何其他的药物治疗，我会事先征求医生的意见。

我同意研究单位上级管理部门、伦理委员会或研究承担单位的代表查阅我的研究资料。

我同意□ 或拒绝□ 除本研究以外的其他研究利用我的医疗记录和病理检查标本。

我将获得一份经过签名并注明日期的知情同意书副本。

最后，我决定同意参加本项研究。

患者签名： 　 日期：＿ ＿ ＿ ＿ 年 ＿ ＿ 月 ＿ ＿ 日

患者联系电话： 手机号：

法定亲属签名： 日期：＿ ＿ ＿ ＿ 年 ＿ ＿ 月 ＿ ＿ 日

法定亲属联系电话： 手机号： 与受试者关系：

我确认已向患者解释了本研究的详细情况，包括其权利以及可能的受益和风险，并给其一份签署过的知情同意书副本。

医生签名： 日期：＿ ＿ ＿ ＿ 年 ＿ ＿ 月 ＿ ＿ 日

医生的工作电话：

中国中医科学院广安门医院伦理委员会办公室联系电话：010-88001552
